# The Use of Nutraceutical and Pharmacological Strategies in Murine Models of Autism Spectrum Disorder

**DOI:** 10.3390/cells13242036

**Published:** 2024-12-10

**Authors:** Matteo Bonetti, Elisa Borsani, Francesca Bonomini

**Affiliations:** 1 Division of Anatomy and Physiopathology, Department of Clinical and Experimental Sciences, University of Brescia, 25123 Brescia, Italy; matteo.bonetti@unibs.it (M.B.); elisa.borsani@unibs.it (E.B.); 2 Interdepartmental University Center of Research “Adaptation and Regeneration of Tissues and Organs (ARTO)”, University of Brescia, 25123 Brescia, Italy; 3 Italian Society of Orofacial Pain (Società Italiana Studio Dolore Orofacciale—SISDO), 25123 Brescia, Italy

**Keywords:** autism spectrum disorder, murine model, therapeutic strategies

## Abstract

Autism spectrum disorder (ASD) is a common neurodevelopmental condition mainly characterized by both a scarce aptitude for social interactions or communication and engagement in repetitive behaviors. These primary symptoms can manifest with variable severity and are often paired with a heterogeneous plethora of secondary complications, among which include anxiety, ADHD (attention deficit hyperactivity disorder), cognitive impairment, sleep disorders, sensory alterations, and gastrointestinal issues. So far, no treatment for the core symptoms of ASD has yielded satisfactory results in a clinical setting. Consequently, medical and psychological support for ASD patients has focused on improving quality of life and treating secondary complications. Despite no single cause being identified for the onset and development of ASD, many genetic mutations and risk factors, such as maternal age, fetal exposure to certain drugs, or infections have been linked to the disorder. In preclinical contexts, these correlations have acted as a valuable basis for the development of various murine models that have successfully mimicked ASD-like symptoms and complications. This review aims to summarize the findings of the extensive literature regarding the pharmacological and nutraceutical interventions that have been tested in the main animal models for ASD, and their effects on core symptoms and the anatomical, physiological, or molecular markers of the disorder.

## 1. Introduction

Autism spectrum disorder (ASD) is a neurodevelopmental disease which is identified and diagnosed by two core symptoms: a scarce tendency for social interactions and engagement in repetitive behaviors. These behavioral symptoms remain today the main criteria used to diagnose ASD, and they start to manifest in children between the second and fourth year of age [1]. Beside core symptoms, these patients show a higher incidence of sleep problems, gastrointestinal issues, altered sensory reactions, anxiety, depression, ADHD (attention deficit hyperactivity disorder), and eating disorders when compared to the general population [2]. Although ASD causes have yet to be fully described, many risk factors for ASD pathogenesis have been discovered, such as genetic background, exposure to some drugs or pathogens during pregnancy, advanced maternal age, and gestational diabetes. These elements have been previously shown to interfere with normal neurodevelopment, causing ASD-linked features such as neuroinflammation, oxidative stress, and mitochondrial dysfunction, imbalance between excitatory and inhibitory neurotransmission (E/I imbalance), and dysregulated synaptic pruning [3]. Therapy-wise, no successful treatment for the core symptoms of ASD has been approved yet, and so far, ASD treatment has focused on managing the secondary symptoms through psychological or educational support and pharmaceutical intervention. In the search for a satisfying therapy for ASD, many preclinical models have been developed and have shown high face value and good potential for the testing and evaluation of new treatments. This process was considerably accelerated by genome-wide association studies, which identified a plethora of ASD-linked gene variants [4], some of which were then replicated in various mouse strains (Table 1). Other ASD murine models are characterized by the idiopathic onset of the disorder, either by complex genetic background, as in the BTBR mouse strain, or as a consequence of fetal exposure to valproic acid (VPA), a drug known to increase the risk of developing ASD in humans when exposed during pregnancy [5]. Murine models currently employed in ASD research are characterized by behaviors, inflammatory profiles, poor gut health, and brain anatomical and physiological alterations that closely resemble those often found in ASD patients. This review aims to briefly list and discuss previous original research articles regarding nutraceutical and pharmacological interventions in preclinical murine models of ASD. More specifically, the review will consider the current literature regarding preclinical original research articles on ASD murine models in which the therapeutic effect on both ASD-like behaviors and on phenotypic manifestations linked to ASD are evaluated.

## 2. Neurotransmission Modulation

Neurotransmission modulation represents a key event in ASD, together with efficient circuitry establishment. They are influenced by the type and amount of neurotransmitters involved, as well as the expression of their receptors, the type and number of active synapses, and thus the neural interconnection [6]. In this context, modulation of neurotransmission may be a possible target for understanding the underlying mechanisms of ASD and, therefore, a therapeutic approach.

### 2.1. Peptides

Duffney and collaborators (2015) [7] behaviorally and molecularly evaluated Shank3+/ΔC juvenile male mice. The Shank3 gene encodes a scaffolding protein at postsynaptic density (PSD) of glutamatergic synapses, which has been demonstrated to be important in ASD behavior, so this is considered a model for this pathology [8]. Indeed, Shank3-deficient mice mimicked the impaired social behaviors typical of ASD, together with a decrease in NMDA-type glutamate receptor (NMDAR) synaptic function and its distribution in the prefrontal cortex [7,9,10]. These deficits were correlated with altered Rac1/PAK/cofilin signaling and dysregulated F-actin in the frontal cortex, which were partially reversed by intraventricular injection of TAT-p-cofilin peptide [7]. These preliminary assumptions potentially link cytoskeleton organization and plasticity at the synaptic level with receptor trafficking and behavioral symptoms, although this association will need subsequent data to be proved.

### 2.2. Pharmacological Compounds

Qin and collaborators (2015) investigated the effect of acute intraperitoneal administration of R-baclofen in an adult male animal model of Fragile X Syndrome (Fmr1 KO mice) [11]. In this model, an imbalance between glutamatergic excitatory signaling and GABAergic (gamma-aminobutyric acid) inhibitory signaling has been described, just as in ASD patients, together with an increased protein synthesis rate in brain tissue. R-baclofen is a selective GABAB receptor agonist and has been successfully used in animal models, with potentially interesting results in a Phase 2 clinical trial [12]; nevertheless, a Phase 3 clinical trial did not show effectiveness in ASD patients, and was consequently ended (as referred to in Qin et al., 2015 [11]). In this research context, Qin and collaborators [11] demonstrated a positive effect of acute R-baclofen in a social behavioral test with a reversed elevated protein synthesis rate in vivo, in particular in the dorsal hippocampus. Interestingly, this study supported the role of the mammalian target of the rapamycin (mTOR) pathway in the regulation of protein synthesis, especially at synapse levels. Finally, a compensatory increase in metabotropic glutamate receptor 5 expression in the frontal cortex could explain the tolerance to the treatment observed in human studies. Also interestingly, in 2021, Möhrle and collaborators administered (acute and systemic) R-baclofen in Cntnap2 KO rat model (female and male) and showed a recovery of ASD-related altered sensory processing caused by E/I imbalance in the auditory brainstem [13]. Additionally, brain amino acid levels in the nucleus reticularis pontis caudalis (PnC) were increased (glutamine, glutamate, and GABA) in the Cntnap2 KO rat when compared to control. Thus, for the authors, this brain area represents the most promising target for R-baclofen (ClinicalTrials.gov ID NCT01352611). Furthermore, Fmr1 KO adult male mice were used to test long term administration of fluoxetine in drinking water (about 10 mg/kg/day), a member of the class of selective serotonin reuptake inhibitor compounds [14], which are antidepressant drugs that are also commonly prescribed to young ASD patients [15]. Fluoxetine inhibited anxiety-like behavior and normalized locomotor activity; moreover, BDNF and TrkB protein expression decreased and increased, respectively, in comparison to the control treatment. On the contrary, fluoxetine did not affect the expression of these molecules in the hippocampus; therefore, they may not be related to the anxiety phenotype. It is noteworthy that the clinical use of fluoxetine is not supported by strong evidence in ASD patients [16], especially considering that its effects have been observed to be highly variable within the ASD patient population, with some notable side effects [17] (ClinicalTrials.gov ID NCT00027404). Very recently, the role of TrkB in young Fmr1^−/−^ mice has been investigated [18] considering the physiological role of BDNF-TrkB signaling for neuronal morphogenesis and synaptic plasticity [19]. The mice (2 weeks old) were intraperitoneally injected daily for 16 days with 7,8-Dihydroxyflavone (7,8-DHF), a high-affinity TrkB agonist, and the results showed a morphological improvement in dendritic spine and synaptic structure and rescued synaptic and hippocampus-dependent cognitive dysfunction, increasing p-TrkB and activating the PLCγ1-CaMKII signaling pathway. These data indicate that early intervention on this target could have future clinical applications. Furthermore, in the same animal model (Fmr1 KO adult male mouse), Liu and collaborators (2011) supplemented the diet with lithium from weaning (21 days) to 12 weeks [20]. Lithium is not considered a micronutrient, even if it is present in foods, and is a mood stabilizer used clinically to treat bipolar disease [21]. Its effects are mediated by several known mechanisms, and among them, the inhibition of glycogen synthase kinase-3 (GSK-3) [22] is considered the principal mediator. Lithium treatment has also been found to partially normalize general anxiety levels and dendritic spine morphology in the prefrontal cortex (ClinicalTrials.gov ID NCT04623398). Additionally, the chronic administration (8–18 days) of sodium bromide (NaBr) has been recently tested by Derieux and collaborators (2022) [23] in three animal models: Oprm1^−/−^, Fmr1^−/−^, and Shank3^Δex13–16^ mice (male and female, aged 8–12 weeks), which showed relieved ASD-like behaviors. In addition, in Oprm1^−/−^ mice, it increased the expression of genes coding for chloride ion transporters, GABAA receptor subunits, oxytocin, and mGlu4 receptors. The results revealed a possible therapeutic use of NaBr alone or in combination with a positive allosteric modulator of the mGlu4 receptor. With regard to Fmr1^−/−^ mice, a link between the Rac/PAK pathway and Fmr1 has been proposed [24]. Dolan and collaborators (2013) presented a PAK inhibitor, called FRAX486, able to modulate ASD-like behaviors and reverse spine phenotype in adult Fmr1^−/−^ mice (aged 10–17 weeks) after acute or chronic treatment [25]. Subcutaneous administration was viable because of the ability of FRAX486 to cross the blood brain barrier. Furthermore, an interesting early correction strategy has been recently proposed with the use of memantine in Shank2-KO mice [26,27]. This animal model showed a decrease in NMDAR function and ADS-like behavior at the juvenile (around day 21) and adult stages, while an opposite trend was observed around day 14, where an increase in NMDAR function was reported. Thus, the early and chronic administration of memantine, at between 7 and 21 days of age, exerted NMDAR modulation but also downstream modulation of gene expression related to chromatin remodeling, mitochondria, and actin. Interestingly, this prevented the onset of ASD-like behavior in the adult stage.

### 2.3. Nutritional Factors

Another point to consider in ASD occurrence surrounds environmental influences and the nutritional factors associated with this pathology. Among them, zinc has been widely discussed, because this trace element has a pivotal role in human health and its deficiency has been associated with ASD [28,29], even if strong supporting data are lacking. In this regard, Lee and collaborators (2015) [30] described the behavioral beneficial effects of clioquinol, a zinc chelator and ionophore, administration (acute and intraperitoneally) in two mouse models, Tbr1^+/−^ and Shank2^−/−^ male mice, with reduced NMDAR function [31,32]. The results of this study suggest that clioquinol enhanced NMDAR function through Src activation [33]. These data are also supported by experiments on ZnT3^−/−^ male and female mice [34], which showed that post-synaptic zinc ameliorated social interaction deficits in the two behaviorally tested mouse models. Additionally, zinc supplementation, both in young mice and during maternal gestation, has been tested and has consistently generated some positive results. Zinc supplementation was tested in young mice for 6 weeks from weaning (postnatal day 21), in particular in Shank3^ex13–16−/−^ male and female mice given a zinc supplementation diet (150 ppm, 30 ppm as control) [35] or in BTBR male mice given a zinc water supplementation (60 ppm) [36]. Additionally, other experiments have tested the potential of prenatal zinc supplementation in ASD prevention in VPA mice (2 mg/kg subcutaneously, 1 h before valproic acid treatment) [37] and a Shank3^−/−^ mouse model (maternal zinc supplementation diet 150 ppm, 30 ppm as control) [38]. In particular, this last work presented interesting results showing that maternal dietary zinc supplementation during pregnancy and lactation protected against the development of ASD associated behavioral and synaptic changes at the glutamatergic cortico-striatal pathway in the offspring and, remarkably, these beneficial effects were maintained into adulthood. The use of zinc in clinical trials has also been reported. Among these reports, a timing-necessity where the early treatment could be a key factor in promoting the normal development of the nervous system, stabilizing future interconnections, which could have a long-term effect in adulthood. Today, the clinical trials performed with some of the described compounds are not satisfactory, to our knowledge, for widespread use.

## 3. Oxytocin

Extensive research has demonstrated a wide role for oxytocin (OXT) in developing and strengthening interpersonal bonding. OXT is mainly synthesized in the hypothalamus at the level of the supraoptic nucleus (SON) and paraventricular nucleus (PVN), and later, it is released into the bloodstream. Then, OXT modulates lactation and uterine contraction during labor, among other physiological properties, while in the central nervous system (CNS), many aspects of human social behavior have been linked to OXT’s action as a neurotransmitter and neuromodulator. Mutations affecting the oxytocin receptor (OXTR) have been linked to ASD core symptoms. Moreover, ASD patients often display significantly reduced levels of serum OXT [39]. Similarly, mice with altered OXTR expression or OXT-related neurotransmission display decreased social preference and are, therefore, commonly employed as ASD animal models [40]. More specifically, OXTR-null mice have been found to show limited social interest and impaired social recognition, together with an increased susceptibility to seizure-causing stimuli; interestingly, this has been paired with normal motor activity and anxiety-like behavior [41]. In these mice, OXT elicited a positive effect on social deficits, which was then neutralized by vasopressin receptor 1a (V1a) blocking. This effect was hypothesized to be caused by the structural similarity of OXT and vasopressin, which allows it to act through vasopressin receptors and yield a similar response in the CNS. Interestingly, the authors showed how vasopressin receptors and OXTR located slightly differently in the CNS [41], suggesting that the V1a-mediated effect may moderately differ from the normal OXTR one and that such differences may emerge in subsequent pre-clinical or clinical studies. Cntnap2 KO leads to, together with ASD-like behavioral symptoms, a significant decrease in OXT immunoreactivity and OXT positive cells in the PVN, which becomes visible a few weeks after birth. Although these symptoms are positively affected by OXT therapy, a notable difference has been determined in OXT chronic administration when treatment begins one week rather than five weeks after birth. These data suggest that endogenous OXT decreases progressively in Cntnap2 KO mice and that there is a timeframe in neural development where intranasal exogenous OXT administration can yield the best results. Interestingly, in these mice, acute OXT treatment was significantly impacted by blocking OXTR, but not V1a, which suggests that OXTR, when expressed, remains the main effector of OXT-mediated behavioral improvements [42]. A recent paper on the same ASD murine model better characterized a neuromodulatory effect of OXT treatment. Here, OXT positively impacted functional connectivity between major brain structures in both limbic and cortical areas, which Cntnap2 KO altered. This study also highlighted the central role of the nucleus accumbens (NAc), where activation of OXTR has been shown to significantly improve social behavior in KO mice. These data suggest that OXT can express a broad neuromodulatory effect and significantly reinforce what has been previously hypothesized to be a network overseeing social reward and behavior [43]. A remarkable study from Hörnberg and colleagues [44] has recently evaluated OXT treatment in a C57BL/6j mouse strain with neuroligin-3 (*Nlgn3*) KO. This gene encodes for a synaptic adhesion molecule previously associated with ASD [45]. These mice showed reduced social recognition, which was linked to reduced firing of oxytocinergic neurons in the ventral tegmental area (VTA), projecting to the NAc. In support of this finding, localized rescue of Nlgn3 KO and OXTR agonist treatment rescued the altered social behavior and reduced firing. Moreover, proteomic analysis of VTA samples found significant alteration in protein levels in pathways linked to molecular adhesion, protein transport and, surprisingly, mRNA translation. Acting on the last pathway, treatment with an inhibitor of MAP-kinase interacting kinases (MNKs) achieved results similar to the OXTR agonist. This suggests a potential link between OXT signaling, mRNA translation regulation, and social recognition, ultimately paving the way for new therapeutic strategies. A new approach has been recently trialed that involves increasing endogenous OXT expression by administering commercially available and slightly neuroprotective molecules. One study used the VPA mice model to determine the potentially neuroprotective effect of maca (*Lepidium meyenii*) extract on ASD symptoms and OXT signaling [46]. Sustained maca extract treatment effectively increased VPA mice performance in social interactions and social memory tests while at the same time increasing c-FOS, a marker of neuronal activity, in key oxytocinergic regions of the brain, such as the SON and the PVN. Other studies have aimed to stimulate endogenous OXT levels by stimulating CD38 expression, a mediator of OXT response, through all-trans retinoic acid derivatives such as beta-carotenoids [47,48]. These studies proved that carotenoids, when given a few days after birth, can ameliorate some behavioral alterations in BTBR mice. These molecules are likely to act by upregulating CD38 in the hypothalamus and the hippocampus with positive repercussions on central brain derived neurotrophic factor (BDNF) and serum OXT levels; however, the precise molecular mechanisms linking this effect with behavioral improvement are yet to be fully characterized. Despite the positive results achieved by some researchers, exogenous OXT in other ASD models has sometimes yielded conflicting results, suggesting that many other factors are relevant to the efficacy of OXT treatment in ASD [49]. Concurrently to preclinical research on OXT, a considerable number of clinical trials employing intranasal OXT have been carried out on many different cohorts of ASD patients. In these trials, OXT was often well tolerated, with adverse effects that did not occur in a significantly different manner between OXT and placebo groups, and with no clear causal link between OXT and the severe events reported [50]. Regarding the efficacy of OXT in the treatment of ASD core symptoms, the current literature does not provide a clear and unambiguous answer, with clinical trials registering a non-significant to a moderately positive response to acute or chronic OXT treatment in ASD patients [51]. Together, the preclinical studies and clinical data suggest further experimentation is needed to evaluate OXT efficacy in ASD properly.

## 4. Antioxidants and Anti-Inflammatory Treatments

Recently, it has been suggested that the adaptive immune response plays a significant role in ASD development [52]. Emerging evidence indicates that altered immune parameters may be implicated in the neurobiological etiology of ASD. Since 1982, it has been known that the immune system impacts cerebral function in ASD; thus, the knowledge regarding this effect in this disorder is not a recent development [53]. In the CNS, many functions typical of peripheral immune cells are taken on by astrocytes and other glial cells. Glial cells also actively contribute to neuron metabolism, differentiation, synaptic plasticity, and impulse transmission. Often, altered development of glial cells or gliosis has been reported in ASD patients [54,55,56] and, even if with less consistency, in ASD murine models [57,58], further suggesting their relevance in ASD pathogenesis. Moreover, a direct link between astrocyte activation and ASD-like behavioral alteration has been reported before [59]. Additional support for the role of immune dysregulation in the pathophysiology of psychiatric disorders comes from studies showing the immunomodulating effects of antipsychotics and antidepressants and the mood-altering effects of anti-inflammatory therapies [60]. Recently, some anti-inflammatory and antioxidant compounds have shown beneficial effects in preclinical models of ASD. These compounds can be administered as single molecules, complex natural extracts, or as synthetic inhibitors.

### 4.1. Antioxidant or Anti-Inflammatory Molecules

Among the single molecules tested in ASD models is icariin (ICA), an active compound extracted from the plants of the genus *Epimedium*, a plant important to traditional Chinese medicine. ICA supplementation (daily for ten days) ameliorated social deficits in the BTBR mouse model of ASD, affecting repetitive stereotypical behaviors and short-term memory deficits without affecting locomotor activity or anxiety-like behaviors in this mice strain. Furthermore, ICA treatment inhibited neuroinflammation and the levels of proinflammatory cytokines in the hippocampus of BTBR mice. Moreover, ICA administration has also been shown to mitigate the disturbed balance of excitatory-inhibitory synaptic proteins, suggesting its role as a novel promising drug for ASD [61]. Wu et al. in 2022 showed the protective effect of selenium (Se) supplementation in BTBR mice [62]. In particular, after selenium administration, the neuronal damage in the hippocampal cornus ammonis (CA1) region of BTBR mice was decreased, the levels of ferroptosis markers significantly declined, and the expression of nuclear factor erythroid 2-related factor 2 (Nrf2), an essential transcription factor that regulates the cellular oxidative stress response, and peroxidation inhibiting protein glutathione peroxidase 4 (GPx4) increased. In addition, the Nrf2 inhibitor, ML385, and the GPx4 inhibitor, RSL3, countered the protective effects of Se, inducing ferroptosis and cognitive and behavioral impairments. These data indicate the protective effect of Se supplementation acting via ferroptosis reduction through increased Nrf2/GPX4 signals in BTBR mice. Moreover, the same group showed improved altered behaviors in BTBR mice after Se supplementation [63]. They supposed that this effect could be associated with the ability of Se supplementation to alter monoamine neurotransmitter levels in brain tissue, inducing oxidative stress reduction and less neuroinflammation in hippocampal tissue, and relieving neural cell damage. Among the anti-inflammatory natural molecules used for ASD treatment, in 2020, Zhong and collaborators tested curcumin administration in BTBR mice [64]. Neonatal curcumin treatment rescued ASD-like behaviors by enhancing sociability, attenuating repetitive behaviors, and improving cognition. Additionally, the curcumin-induced behavioral changes were paired with the promotion of hippocampal neurogenesis. Similarly, hippocampal inflammation and decreased neurogenesis can be restored using minocycline treatment. In two separate studies on two different ASD models, minocycline positively regulated microglial activation and proliferation, with positive repercussions for ASD-like behavior [65,66]. These data underline the already well-documented correlation between altered microglia phenotype, neurodevelopment, and ASD. Bakheet et al. (2016) studied the effect of resveratrol on the production and expression levels of CC-chemokine receptor 5 (CCR5), a member of the CC-chemokine receptor family. The authors showed how resveratrol treatment decreased CCR5 expression in the spleen cells BTBR mice [67]. In line with these observations, the group showed that resveratrol treatment inhibited CCR5 mRNA expression in the spleen and brain tissues. The inhibitory effects of resveratrol were also confirmed in the B6 mice strain, which exhibited a significant decrease in CCR5 receptor expression. These data suggest that the suppressive effect of resveratrol on CCR5 receptor production might be useful in ASD by modulating the immune response through chemokine signaling. In the same animal model, Borsani et al. in 2022 [68] further reported how an anti-inflammatory and antioxidant molecule, such as melatonin, can restore the antioxidant response likely compromised in BTBR mice. Melatonin was also shown to improve the GABAergic/glutamatergic balance and modulate neural plasticity markers. However, 8-week melatonin treatment at the dose considered could not significantly restore the behavioral alterations in BTBR mice. These results may be due to limited treatment duration, dosage, or sample size. In the same study, melatonin administration promoted the expression of antioxidant enzymes, confirming the key role of melatonin against oxidative stress, which is common in ASD patients. In a follow-up study, these observations were confirmed in the hippocampus, where a beneficial effect of melatonin treatment against ferroptosis, inflammation, and oxidative stress was reported [69]. Another study by Tian et al. (2014) [70] investigated the effects of melatonin in two experimental ASD models by examining the expression levels of key protein kinases involved in learning and memory. Disruptions in hippocampal levels of Ca^2+^/calmodulin-dependent protein kinase II (CaMKII), Ca^2+^/phospholipid-dependent protein kinase C (PKC), and cyclic AMP-dependent protein kinase A (PKA) signaling were observed in both VPA-treated rats and BTBR mice. Importantly, melatonin significantly prevented the hypophosphorylation of serine/threonine kinase signaling and the decrease in long-term potentiation (LTP) in the hippocampus of the ASD models. These data suggest that melatonin, in addition to neuroprotection, exerts beneficial effects on E/I imbalance and on CaMKII/PKC/PKA phosphorylation, and induces LTP, which ultimately correlates with the ameliorations in social behavior dysfunction observed in ASD murine models. In the VPA model, Yadav et al. (2017) [71] studied the effect of two anti-inflammatory polyunsaturated fatty acids (PUFAs), in particular alpha-linolenic acid (ALA, 18:3 n3) and gamma-linolenic acid (GLA, 18:3 n6), which are paramount for humans due to their wide array of role in physiological systems, including the developing brain. ALA and GLA treatments restored the rate of weight gain in VPA mice. Moreover, the results showed that ALA and GLA could impart favorable protection against VPA-induced ASD-like features, such as cerebellum neuronal loss and microglial activation, potentially via the treatment’s antioxidant and anti-inflammatory action. In the same year, Al Amin and collaborators (2015) studied the effects of astaxanthin, a well-known antioxidant, in VPA mice [72]. They showed that this molecule, known to cross the blood–brain barrier (BBB), together with exhibiting antioxidant and neuroprotective properties, also improved ASD-like behaviors. Moreover, it improved antioxidant response while decreasing lipid peroxidation markers, such as brain malondialdehyde (MDA) levels. Zamberletti and collaborators (2019) hypothesized the involvement of altered endocannabinoid signaling and immune dysfunction in ASD pathogenesis [73]. Therefore, they evaluated the effect of the phytocannabinoid cannabidivarin in VPA mice. This phytocannabinoid in symptomatic rats successfully recovered behavioral impairments. Moreover, cannabidivarin restored, at the neurochemical level, hippocampal endocannabinoid signaling and sensibly lowered neuroinflammation induced by prenatal VPA exposure. Poleg et al. (2021) investigated another cannabinoid molecule, cannabidiol (CBD), on behavior and biochemical markers in a well-characterized genetic mouse model of ASD in Shank3 mice [74]. The authors demonstrated a reduction in repetitive grooming and anxiety behaviors after treatment with CBD-enriched Avidekel oil and highlighted the involvement of cannabinoid receptor 1 and glutamate in this process. Furthermore, in the same study, RNA seq analysis results indicated changes in the expression of neurotransmission and ASD-related genes. Notably, the results of this study suggest relatively high doses of CBD, such as those found in medical cannabis oils, for relieving ASD core symptoms. Moreover, the use of medical cannabis oil with a 1:20 Δ9-tetrahydrocannabinol (THC) to CBD ratio resulted in an additional effect on social behavior and was a preferable treatment in this setting. In 2023, Mehra and coll. analyzed the effect of fisetin, a highly lipid-soluble bioflavonoid, in treating VPA mice [75]. In this study, gestational and post-weaning fisetin administration were tested for VPA-induced behavioral alterations and oxidative stress. The results of this study reported that both gestational and post-weaning treatments with fisetin in VPA mice significantly declined the up-surged lipid peroxidation and regulated the levels of antioxidant enzymes (catalase, SOD, GST, GSH), making cells better equipped to adapt to oxidative stress and ameliorated ASD-like behavior. In 2021, Luhach et al. assessed the effects of papaverine administration on behavioral phenotypes associated with ASD in VPA rats [76]. The results of this study indicated that papaverine induced an amelioration of ASD-linked behavioral deficits, increasing anti-inflammatory marker levels and decreasing oxidative stress in different important brain areas. The same group [77] also evaluated the effect of vinpocetine, a neuroprotective molecule clinically used to prevent cerebrovascular disease and dementia, on the same animal model. The results indicated that administration of vinpocetine improved ASD-associated behaviors, with an improvement in cell viability in the brain’s dentate gyrus (DG) area. The mechanism of action, similarly to those of the papaverine result, might be linked to the antioxidant and anti-inflammatory effects exerted by this molecule. Pragnya et al. in 2013 [78] evaluated the effects of piperine, a major alkaloid present in black pepper (*Piper nigrum*) and long pepper (*Piper longum*) traditionally used in Ayurvedic medicine for different conditions. Piperine is a neuroprotective molecule, given its antioxidant, anxiolytic, and cognition-enhancing effects. In this study, treatment with piperine in the VPA model ameliorated behaviors and positively regulated serotonin levels and oxidative stress. Furthermore, this molecule ameliorated VPA-induced histopathological alterations in the cerebellum. Recently, Jiang et al. (2023) used a flavonoid monomer which can cross the BBB, puerarin, to treat the core symptoms of ASD in VPA mice [79]. The results showed that puerarin supplementation rescued inhibited hippocampus neurogenesis in the VPA mice, further suggesting that boosting early neurogenesis could benefit ASD-like behaviors. Moreover, puerarin treatment inhibited iron overload and lipid peroxidation accumulation and enhanced the expression of ferroptosis-inhibitory proteins in the hippocampus of the VPA mouse model of ASD. In 2022, Abbasalipour et al. used nanophytosomes containing gallic acid or sumac (*Rhus L.*) extract to evaluate the therapeutic potential and antioxidant activities on hippocampal damage in VPA rats [80]. This study’s results proved that using these nanophytosomes significantly improved recognition memory deficits and reduced hippocampal oxidative stress in this ASD animal model. This effect was likely due to the enhancement of the Nrf2 pathway, further reinforcing the central role of this enzyme in antioxidant response and its relevance in ASD murine models. Furthermore, a study by Mallan and Singh (2023) employing syringic acid in VPA rats further showed how reducing inflammation and oxidative stress can positively impact E/I imbalance and ASD behavioral symptoms [81].

### 4.2. Natural Extracts

Plant extracts are a renowned source of bioactive compounds, notably polyphenols and terpenoids, that have been screened in animal models as protective molecules against many diseases, including ASD. In 2012, Sandhya et al. evaluated the effect of *Bacopa monniera*, a plant commonly used in Ayurvedic medicine for neurological disorders, on VPA-induced ASD [82]. The authors demonstrated that fetal VPA treatment significantly affected social behavioral performances, increased oxidative stress and SNC serotonin levels and caused morphological alterations at the CNS level. Such changes were individuated in cerebellar histoarchitecture with a decreased Purkinje cells number and neuronal degeneration. Treatment with *B. monniera* extract significantly improved animals’ social behavior and decreased catalase and glutathione levels in the brain while restoring Purkinje cell density. These results potentially showed that restoring physiological oxidative stress can positively affect ASD symptoms by restoring cerebellar cell density. Similarly, purple rice (*Oryza sativa*) and pupae of silkworm (*Bombyx mori*) extract have been shown to possess antioxidant and neuroprotective effects. This extract yielded similar results regarding oxidative stress, social behavior, and Purkinje cell density in VPA rats [83]. Arafat et al. in 2019 further examined the cerebellar alteration caused by fetal VPA treatment in rats and considered the effect of grape seed extract, a natural and rich source of phenolic compounds, especially flavonoids [84]. In the VPA rat model, the authors observed a significant decrease in enzymatic protection against oxidative stress, in addition to astrocyte hyperactivation and cerebellar gliosis, which were associated with loss of Purkinje cell density, likely via neuronal cell death. Grape seed extract administration showed a potent antioxidant effect, protecting the cerebellum from oxidative damage and normalizing the alteration caused by fetal VPA administration. Conversely, many researchers have focused on the effect of similar plant extracts in the hippocampus of ASD murine models, another CNS region strongly correlated with memory and social behavior development. Park et al. (2021) evaluated the effect of a *Humulus japonicus* extract in a different ASD murine model, the BTBR mouse [85]. This traditional herbal medicine has demonstrated neuroprotective properties. When chronically administered, this extract has been shown to significantly ameliorate behavioral deficits and neuroinflammation, evaluated by cytokine chemokine (C-C motif) ligand 2 (CCL2) expression in the BTBR mice hippocampus. Recently, in the same CNS region, Hussein et al. (2023) studied the possible effect of palm date aqueous fruit extracts (AFE) on autistic-like behaviors and oxidative stress in VPA rats [86]. In particular, the authors evaluated behavioral tests as well as oxidative stress markers and the expression of Nrf2, heme oxygenase (HO-1), sirtuin 1 (Sirt-1), the marker of apoptosis caspase-3s, the marker of autophagy microtubule-associated protein 1 light chain 3 beta (LC3b), and the inflammatory cytokine nuclear factor kappa B (NFkB) in brain tissues. The results showed that AFE administration significantly improved autistic-like behavioral alterations and attenuated oxidative stress, as well as upregulating the expression of LC3b, Nrf2, HO-1, and Sirt-1, accompanied by a downregulation of caspase-3 and NFκB expression in the cerebellum and hippocampus. In the same year, Amini et al. (2023) studied the effects of the hydroalcoholic extract of *Passiflora incarnata* administration on behavioral and hippocampal alterations in the VPA rat model [87]. This extract shows potent antioxidant activity due to its high flavonoid, phenolic compound, and cyanogenic glycoside content [88]. In this study, it was found that *Passiflora incarnata* extract significantly increased antioxidant enzyme levels and decreased oxidative stress marker levels in the ASD model. Moreover, it partially decreased the number of damaged neurons in the prefrontal cortex and CA1 region of the hippocampus. Furthermore, administration of the extract rescued the altered behavior, decreasing repetitive activities and stereotyped movements. In addition, Saadat et al. (2023) investigated the effect of *Prangos ferulacea*, a plant largely used in traditional medicine in several countries that has been shown to possess biological properties [89]. It has been tested on behavioral alterations, hippocampal oxidative stress markers, and apoptotic deficits in the same VPA rat. The study results demonstrated that *Prangos ferulacea* administration reduced the neuronal loss in the hippocampus’s CA1, CA3, and DG subregions, probably reducing the elevated oxidative stress by increasing antioxidant enzyme activity in the hippocampus. Moreover, it restored B-cell lymphoma-2 (BCL-2) and BCL2-associated X (Bax) levels, confirming its antiapoptotic effect on pyramidal neurons in the hippocampus of VPA-induced animals. Seyedinia et al. in 2023 also studied the effect of saffron (*Crocus Sativus*) and its active ingredient, crocin, in a VPA-induced rat model of ASD [90]. The study demonstrated that treatment with saffron extract and crocin improved the VPA rats’ brain oxidative stress parameters and ameliorated ASD-associated behaviors. such as increased pain responses, anxiety, and motor and balance deficits. Taken together, these findings highlight the beneficial effect of herbal-based drugs in ASD animal models.

### 4.3. Synthetic Cytokines Inhibitors

Immune abnormalities in ASD are often displayed as increased immune cell activation and an imbalance between proinflammatory/anti-inflammatory cytokines [91,92]. These persistent immune dysfunctions can usually be a contributory cause of behavioral alterations in ASD; this notion is reinforced by the higher incidence of ASD following maternal immune activation and following certain maternal infections [93]. For this reason, some authors have evaluated the effects of some synthetic compounds targeting cytokine receptors or their signaling in animal models of ASD. Among these compounds are transducer activators of transcription 3 (STAT3) inhibitors. STAT3 phosphorylation has been demonstrated to be increased in ASD with, consequently, the promotion of a proinflammatory response [94]. In particular, among STAT3 inhibitors, Ahmad et al. evaluated the effect of S3I-201, which specifically inhibits STAT3 DNA-binding activity and reduces STAT3 phosphorylation [95,96]. These studies showed that S3I-201 treatment led to a significant reduction in neuroinflammation markers, chemokine receptors, and inflammatory cytokines, and to suppression of Th17-related signaling, while enhancing Treg-related signaling. These were linked to improvement in ASD-like behavior in this mice strain. The same research group also evaluated, in the same animal model, the effect of the protein tyrosine kinase inhibitor, tyrphostin AG126 (AG126), which actively regulates the expression of several genes that play a significant role in the pathophysiology of different inflammatory diseases. This molecule showed a potential therapeutic effect in ASD by reducing repetitive behaviors and pain sensitivity in BTBR mice. This effect could be due to the blockade of multiple signaling pathways, such as Th17/Th1 related signaling and the NF-κB pathway in the CNS. Another group individuated, as a potential therapeutic target in ASD, the C-X-C motif chemokine receptor 2 (CXCR2) [97], a G-protein coupled receptor of neutrophils and T lymphocytes essential for cerebral endothelial activation and leukocyte recruitment during inflammation. To test this, they employed the CXCR2 antagonist SB332235. This administration was able to ameliorate behavioral deficits and the dysregulation of Th1/Th22 and Treg cell-related transcription factor signaling in the BTBR mice. This study’s results proved that SB332235 has beneficial and protective effects on ASD-like behaviors and molecular alterations in the spleen and brain tissues. This was likely due to Th1/Th22/Treg related signaling regulation and consequential normalization of the immune response.

## 5. Therapeutic Approaches Targeting the Microbiome

The human gut microbiome comprises the heterogeneous communities and strains of microorganisms in a single human gastrointestinal system. Extensive research has shown how these populations can influence gut health, the CNS, and vice versa. Dysbiosis, appearing as a significant alteration in microbial population equilibrium, has been implicated in many diseases, including neurodevelopmental disorders such as ASD [98]. Moreover, ASD patients report gastrointestinal issues more often than the general population, and dysbiosis is a common finding in these patients. Ultimately, recent studies have suggested that a substantial contribution to ASD symptom severity may be related to dysbiosis. This hypothesis was tested by transferring ASD human patients’ microbiome into otherwise healthy mice through fecal transplant (FT), which resulted in a visible worsening of the cytoarchitecture and cellular vitality biomarkers in the intestine of FT mice, together with behavioral alterations similar to those shown by VPA-treated mice [99]. This symptomatology was paired with decreased overall DNA methylation levels and neuroinflammation, both of which are well-known contributors to ASD etiology. Coherently, the microbiome from healthy mice can rescue behavioral symptoms, gut health, and serum metabolic profile, and can positively influence the expression of genes regulating neurotransmission in the brain of ASD mice [100].

### 5.1. Dietary Interventions

**Figure 1:**
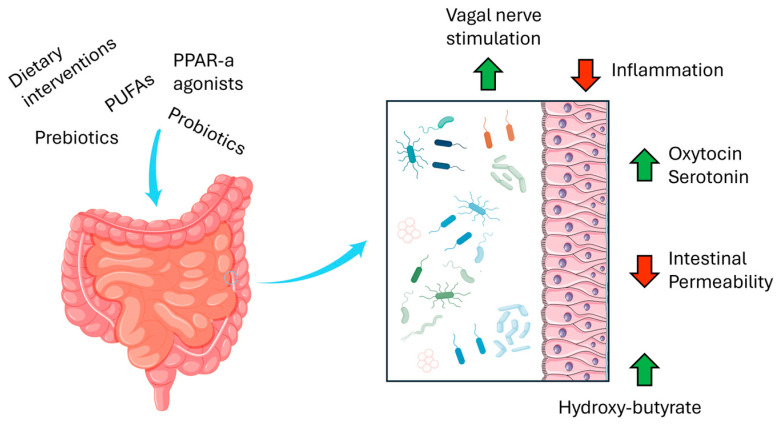
Summary of therapeutic approaches targeting the microbiome and the gut–brain axis. Abbreviations: PPAR-a, peroxisome proliferator-activated receptor alpha; PUFAs, polyunsaturated fatty acids.

Many murine models for ASD organically develop gut inflammation, gut permeability [101], or dysbiosis, suggesting that these are frequent and relevant features of the disorder. More specifically, bacterial communities’ heterogeneity and the ratio between the most common phyla in the gut microbiome, *Firmicutes* and *Bacteroidetes*, are often unbalanced. Many factors, such as host ethnicity and genetics, body composition, antibiotic use, diet, and many environmental factors can influence the human gut microbiome populations. Therefore, patient diet changes are an appealing tool for therapeutic interventions for dysbiosis (Figure 1). In ASD murine models, this approach has led to some early work which explored modulating macronutrient (i.e., carbohydrates, protein, and fat) relative intake and variety. Notably, recent research has focused on PUFAs since different n6 to n3 PUFA ratio (n6/n3 ratio) may significantly impact systemic inflammation and gut health. A diet characterized by a n6/n3 ratio of 1:5 was able to mitigate dysbiosis in VPA-treated mice by normalizing the relative abundance of various phyla and genus and the *Firmicutes/bacteroidetes* ratio [102]. This was paired with the rescue of intestinal permeability, which then likely regulated serotonin absorption and, therefore, serum and CNS levels [103]. In BTBR mice, a low-glycemic index diet during pregnancy and early life led to the normalization of gut microbiome-associated metabolites, behavioral test performances, microglia activation, and hippocampal alterations [104]. Similarly, a ketogenic diet protocol in VPA mice led to the amelioration of ASD-linked behavioral alterations and of mitochondrial function in the neocortex, likely through the normalization of beta-hydroxybutyrate, a known gut microbiome metabolite [105]. Subsequent studies focused on this metabolite, whose beneficial effects could also arise from its known potential as a histone deacetylase (HDAC) inhibitor (HDACi). In Shank3 mice, the ketogenic diet has been linked to increased histone acetylation of NMDAR subunit promoters in the prefrontal cortex, which was linked to increased sociability registered in the treated group [106].

### 5.2. Prebiotics, Probiotics and Postbiotics

Treatment with exogenous sodium butyrate (NaB), a microbial product, has been reported to improve dendritic spine density in the VPA mice hippocampus [107] and to upregulate various genes involved in maintaining the E/I balance in the prefrontal cortex of BTBR mice [108] while ameliorating behavioral symptoms in both VPA and BTBR mice. NaB can also improve ASD-linked repetitive behaviors and low sociability in BTBR mice offspring when supplemented during pregnancy; this has also resulted in improved excitability and plasticity of Purkinje cells at 1 and 2 months of age [109]. Besides dietary interventions, the gut microbiome can be regulated by supplementing live bacterial populations as probiotics. In VPA mice, long-term supplementation with three different *lactobacillus* strains improved ASD behavioral symptoms comparably to risperidone, an FDA-approved drug. Furthermore, these probiotics had a positive effect on microbiome heterogeneity, as measured by the alpha diversity index, and normalized the *Firmicutes/Bacteroidetes* ratio while also lowering the abundance of a serotonin-consuming strain, *Turicibacter sangunis.* This seems to be responsible for *Lactobacillus helveticus’* positive effect on serotonin levels in the colon, feces, serum, PFC, and cerebellum. Moreover, all *lactobacilli* significantly affected the glutamate to GABA ratio in the CNS [110]. In VPA mice, *Lactiplantibacillus plantarum* grown in fermented milk had similar effects on the gut *Firmicutes/Bacteroidetes* ratio, with positive repercussions on ASD-like behaviors [111]. In BTBR mice, the incorporation of two different strains belonging to *Lacticaseibacillus rhamnosus* and *Ligilactobacillus salivarius* in BTBR mice microbiome composition achieved positive effects on behavioral symptoms, and many butyrate-producing bacteria strain were upregulated; conversely, no improvement was reported in mitochondrial health in the CNS, and conflicting results emerged regarding the inflammatory profile induced by the treatment, where both inflammatory and anti-inflammatory cytokines were upregulated by the probiotic treatment with *L. rhamnosus* [112]. The OXT system has been reported also to be upregulated by probiotic supplementation in BTBR, Shank3, and VPA mice. In recent studies, these mice strains have been characterized by the low abundance of, among others, *Lactobacillus reuteri* (*L. reuteri*) [113] in the gut, and supplementation with these strains improved ASD-like behavioral deficits. Remarkably, *L. reuteri* achieved this independently of other gut microbial species by vagal nerve mediation of dopaminergic neurons in oxytocin-sensitive CNS regions, such as the VTA and the NAc [114]. Interestingly, in a recent study conducted in BTBR mice, unclear behavioral results paired with positive data on overall gut health emerged from the combination of prebiotic and probiotic treatment [115], suggesting further research is needed regarding the specific molecules and microbial strains involved.

### 5.3. Anti-Inflammatory Molecules

Downregulation of systemic or gut inflammation has been a promising strategy mitigating ASD-linked behaviors and dysbiosis in these preclinical models. In this perspective, maternal and pre-weaning melatonin supplementation has been experimented with in VPA mice, with positive effects on ASD-like behaviors, microbiome composition, and dopaminergic neuron activity in the VTA. Interestingly, *Akkermansia muciniphila* (*A. muciniphila*) was significantly more abundant after maternal melatonin supplementation, and a similar effect was observed when postnatally treated with *A. muciniphila* alone. Furthermore, subsequent experiments involving the specific inhibition of dopaminergic neurons in the VTA suggest a causal role in part of the improvement seen with early life melatonin or *A. muciniphila* supplementation [116]. Palmitoylethanolamide (PEA), a peroxisome proliferator-activated receptor alpha (PPAR-a) agonist, has been studied for its anti-inflammatory properties in BTBR mice, showing that it improves the hippocampal inflammatory profile and mitochondrial function and reactivates BDNF signaling in the hippocampus [117]. This effect was paired by normalization of ASD-like behaviors, gut permeability, and the microbiome. Interestingly, in PPAR-a KO mice, or when a PPAR-a inhibitor is co-administered to PEA, no significant behavioral improvement is registered in BTBR mice, highlighting the importance of this enzyme. Recently, anthocyanin-rich extract supplementation in VPA has been reported to improve the gut microbiome by increasing the relative abundance of *Lactobacillales* and reducing that of *Clostridales* in VPA mice. This has been linked with a slightly positive impact on sociability in mice, lower gut and CNS inflammation, and a significantly improved ratio for excitatory synaptic transmission in the CNS [118]. When considered together, the studies reported in this section strengthen the hypothesis of a close and reciprocal crosstalk between gut health and the CNS in ASD pathogenesis. These studies highlight the potential of the gut–brain axis as target for both the gastrointestinal and core symptoms reported in ASD patients.

## 6. Targeting mTOR Pathway

**Figure 2:**
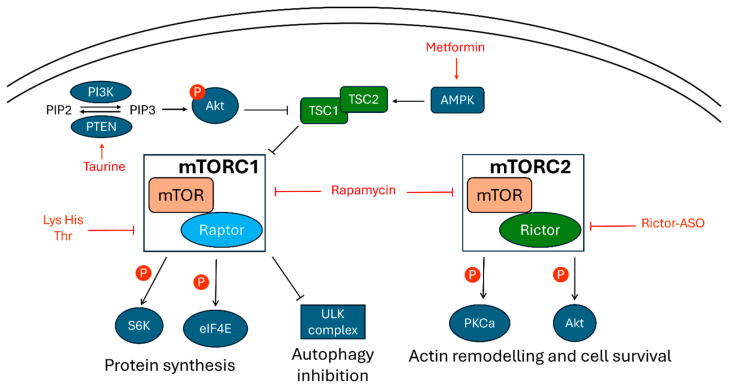
Schematic representation of key elements of the mTOR pathway and of therapeutic interventions considered in murine models for ASD. Abbreviations: PIP2: phosphatidylinositol 4,5-bisphosphate PIP3: phosphatidylinositol 3,4,5-bisphosphate PI3K: phosphatidylinositol 3-kinase; PTEN: phosphatase and tensin homolog; Akt: protein kinase B; TSC1: tuberous sclerosis 1; TSC2: tuberous sclerosis 2; AMPK: AMP-activated protein kinase; mTOR: mammalian target of rapamycin; mTORC1: mTOR complex 1; mTORC2: mTOR complex 2; S6K: Ribosomal protein S6 kinase beta-1; eIF4E: eukaryotic Initiation Factor 4E; ULK complex: Unc-51-like kinase 1 complex; PKCa: protein kinase C alpha; P: phosphate group.

mTOR is a serine/threonine kinase that interacts with various elements to form mTOR complex 1 (mTORC1) and mTORC2. Although these complexes share some components, they can respond to different stimuli and impact diverse cellular responses. mTORC1 is linked to cell growth given its function as a promoter of protein synthesis, ribosomal proteins expression, and the synthesis of purine and lipids. Conversely, mTORC2 mainly regulates cell remodeling and survival through the phosphorylation of protein kinase B, mostly known as Akt, and PKC. Recently, many lines of research have linked dysregulation of the mTOR pathway to ASD (Figure 2). Some studies on ASD patients have observed a hyperactivation of the mTOR pathway at both the systemic level [119] and in the CNS [120] when compared to healthy individuals. Patients affected by neurodevelopmental disorders characterized by genetic activation of the mTOR pathway, such as tuberous sclerosis [121] and fragile X syndrome [122], also show ASD-like symptoms. Similarly, in mice, genetically upregulating mTOR signaling by mutating proteins involved in its pathway, such as phosphatase and tensin homolog (PTEN), results in ASD-like features and behaviors [123]. Therefore, many research groups have considered mTOR inhibition as a promising strategy in ASD treatment. Threonine, histidine, and lysine have been reported to inhibit mTOR successfully; this led Wu [124] and colleagues to explore the effect of a diet rich in these amino acids on BTBR mice and to compare its effects with both a standard diet and a low-glycemic index diet, already known to have a slight neuroprotective effect. This mTOR-active diet significantly inhibited ribosomal protein S6 kinase beta-1 (S6K) phosphorylation, a downstream target of mTOR, in the prefrontal and somatosensory cortex and outperformed the neuroprotective diet in mitigating excessive grooming behavior in BTBR mice [124]. Interestingly, taurine supplementation was able to inhibit Akt and mTOR phosphorylation through PTEN upregulation in the hippocampus of BTBR mice, which led to a positive effect on hippocampal neurogenesis and ASD-like behavioral symptoms [125]. Rapamycin is a strong FDA-approved inhibitor of mTOR, and its positive effect on ASD syndrome has been reported in many ASD murine models. In two cohorts of young and adult VPA rats, rapamycin normalized social behavior in both mice groups [126], and subsequent assays on brain gene expression characterized the genes impacted by VPA in-utero exposure and by rapamycin acute treatment, allowing the authors to hypothesize underlying gene interactions. In Cntnap2-deficient mice 2-day rapamycin treatment rescued social deficit while no significant improvement was registered in repetitive behaviors; notably, this improvement was lost when behavioral tests were repeated 7 days later, suggesting that the positive effect of acute rapamycin treatment is temporary and that chronic treatment may be necessary [127]. In PTEN mice, a 5 day-long rapamycin treatment was able to prevent macrocephaly in a younger cohort and to reverse it in a symptomatic older cohort [128]. Coherently, rapamycin successfully treated neuronal hypertrophy in the cortex and in the hippocampus, in the latter, it also impacted mossy fibers and DG morphology and dendritic spine density and size. This positively affected ASD-like behavior, as it significantly increased total interaction time in the reciprocal social interaction test. These DG neuron dendritic morphology results were successfully replicated by a longer treatment in a cohort of younger PTEN mice. Interestingly, these improvements were maintained in a double knock-out (DKO) for PTEN and Raptor, a protein present in the mTORC1 but not in mTORC2 complex, and lost in a DKO for PTEN and Rictor, an exclusive component of mTORC2. This ultimately suggested that the benefits of rapamycin treatment derive from the inhibition of mTORC1 rather than mTORC2 [129]. This hypothesis is reinforced by other studies employing metformin, a molecule which inhibits mTORC1 via AMPK, as a potential treatment for ASD symptoms. In Fmr1 mice, metformin treatment starting at birth rescued repetitive self-grooming behavior while simultaneously normalizing the phosphorylation level of mTOR downstream targets, important kinases like mitogen-activated protein kinase kinase (MEK) and the extracellular signal-regulated kinase (ERK), and the expression level of metalloprotease 9 [130]. Similarly, metformin treatment achieved comparable results in mice deficient for Bmal1, a gene known to be the central gene for circadian rhythm regulation. This murine model, besides repetitive behaviors and impaired sociability, was also characterized by altered motor learning. The authors hypothesized that these symptoms were due to an alteration in cerebellar development following Bmal1 KO. This was then visible from a significantly higher fraction of immature dendritic spines in Purkinje cells, which had consequences for both excitatory and inhibitory neurotransmission. The Bmal1 KO mice cerebellum showed a significant difference in the expression of several ASD-linked genes, among which were identified networks involving multiple genes related to circadian rhythm regulation, important genes for neural cell development such as *Ntng2*, *Nr4a2*, and *Thbs1*, and multiple genes that play a part in the eukaryotic initiation factor 2, 4E (*eIF2*, *eIF4E*), and S6K pathways, suggesting a hyperactivation of mTORC1 pathway. Through its action as a mTORC1 inhibitor, metformin rescued neurotransmission in Purkinje cells and improved social and self-grooming behaviors, ultimately suggesting an important role for mTORC1 in the Bmal1 KO model of ASD. Surprisingly, a different study brought strong evidence suggesting that mTORC2 is more relevant to ASD pathogenesis than mTORC1 [131]. This study initially showed how, in the hippocampus and prefrontal cortex of PTEN mice, both mTORC1 and mTORC2 downstream targets are over-phosphorylated, suggesting activation of both complexes. Therefore, the authors employed DKO constructs for the silencing of either mTORC1 or mTORC2 in PTEN mice with the aim of evaluating the specific impact of both complexes. The use of PTEN and Raptor DKO rescued the macrocephalic phenotype typical of PTEN mice, which was not achieved with PTEN and Rictor DKO, suggesting that mTORC1 overactivation has a central role in the development of that phenotype. Conversely, only PTEN and Rictor DKO partially rescued ASD-like behaviors, reduced sensitivity to seizure-inducing stimuli, and reversed the increased excitatory synaptic transmission in the CA1 region of the hippocampus. This strongly suggests that mTORC2 overactivation is responsible for most of the ASD-linked symptoms observed in PTEN mice. This was further confirmed by the in vitro and in vivo experiments conducted using single-strand antisense oligonucleotide (ASO) targeting Rictor mRNA, which yielded similar results to the PTEN and Rictor DKO, paving the way for a different approach and strategy for ASD treatment. Taken together, these studies suggest the importance of mTOR hyperactivation in ASD pathogenesis and, consequently, its potential as a therapeutic target. Unfortunately, these studies are not resolutive regarding which among the mTOR interactions should be targeted. These conflicting results may be explained by the heterogeneity of mTOR interactions in different cell types and steps of neurodevelopment, and by the different models and tools employed for their characterization.

## 7. Epigenetic Modulation

**Figure 3:**
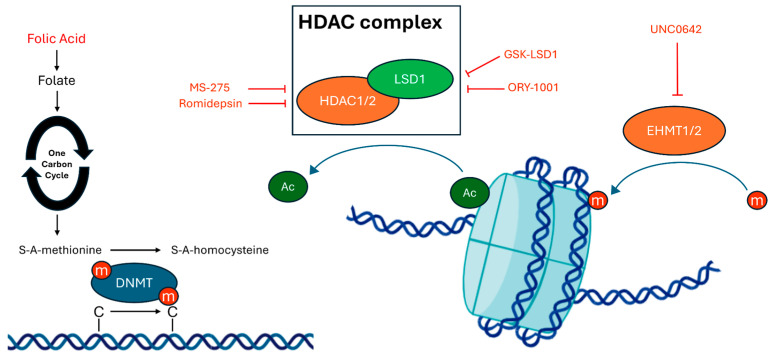
Schematic representation of epigenetic modifications targeted by therapeutic interventions in murine models for ASD. Abbreviations: HDAC complex: histone deacetylase complex, HDAC1/2: histone deacetylase 1 and 2, LSD1: lysine-specific demethylase 1A; EHMT1/2: eurochromatic histone-lysine methyltransferase 1 and 2; DNMT: DNA methyltransferase, m: methyl group; Ac: acetyl group.

Epigenetic modifications, such as histone methylation and acetylation, have a strong effect on chromatin assembly, structure, re-modelling and, therefore, gene expression. Recently, altered methylation and acetylation profiles have been linked to several diseases, including many neurodevelopmental disorders. Postmortem studies on ASD patients have detected altered epigenetic modifications [132,133], and simultaneously, genome-wide associations have been made between DNA methylation and ASD [134,135]. Folic acid (FA) has a central role in the one-carbon cycle, a biochemical pathway that supports DNA synthesis, repair, and methylation. For its importance in neural development, FA supplementation has been previously explored as a potential treatment for ASD core symptoms in ASD murine models (Figure 3). In BTBR mice, post-weaning FA supplementation has significantly lowered neuroinflammatory markers in the hippocampus while upregulating the activity of key enzymes for the oxidative stress response and iron metabolism. Ultimately, FA has displayed overall neuroprotective activity with positive repercussions on mice sociability indexes and repetitive behaviors [136]. Subsequently, maternal FA supplementation showed similar behavioral improvements in VPA rats, which was coupled with the return to control levels of synaptic activity markers and dendritic spine density in the prefrontal cortex [137]. Recent research has developed a more direct way of impacting epigenetic profiles through small molecules able to inhibit the enzyme responsible for histone modifications directly (Figure 3). Some of these molecules, when able to cross the BBB, have been recently employed for the treatment of ASD-related symptoms in Shank3-deficient mice. MS-275 (Entinostat), a HDACi, was able to rescue the low level of histone 3 (H3) lysine 9 acetylation (H3K9Ac) in the prefrontal cortex (PFC) of Shank3-deficient mice. Moreover, subchronic administrations of HDACi induced a lasting improvement in sociability, but not in repetitive behaviors, 11 days after treatment. This was associated with the normalization of NMDAR subunits synaptic localization, likely due to the restoration of NMDAR trafficking through the upregulation of the Rac1-PAK-cofilin pathway [138]. Similar results were obtained through a 3-day treatment with romidepsin, another brain-permeable HDACi [139]. In this setting, increased H3K9Ac outperformed other antipsychotics currently approved for ASD treatment in restoring normal sociability, but failed to correct repetitive behaviors in two different Shank3-deficient strains. This effect was likely due to the restoration of NMDAR-mediated transmission in PFC pyramidal neurons. RNA sequencing (RNA-seq) experiments allowed the authors to hypothesize the central role of the cytoskeleton-mediated transport of NMDAR subunits in the positive effect of HDACi. The positive effect of HDACi was then validated in a similar setting involving mice harboring the 16p11.2 deletion, a different mouse model for ASD, where LMK235 [140], MS-275 and romidepsin [141] achieved similar improvement in sociability by normalizing NMDAR-mediated and GABAergic neurotransmission balance in the PFC. Subsequently, other experiments were conducted targeting histone methylation to elicit similar improvement in ASD symptoms by inhibiting histone methyltransferases and demethylase. Altered histone 3 methylation was targeted in Shank3 mice by UNC0642, a brain-permeable small molecule which can act as an inhibitor for both euchromatic histone-lysine methyltransferase 1 (EHMT1) and 2 (EHMT2). This treatment restored normal histone 3 lysine 9 di-methylation (H3K9me2) in the PFC with positive consequences on social parameters and body weight, while motor coordination was not altered by the compound. Furthermore, these effects were likely mediated by the restored NMDAR-mediated currents in the PFC, of which rescue was caused by the recovery of physiological levels of activity-regulated cytoskeleton-associated protein (ARC) [142]. Similar results were achieved in Shank3 and Cullin 3 KO (Cul3) mice through the inhibition of Lysine-specific histone demethylase 1A (LSD1) [143]. Here, treatment with both GSK-LSD1 and ORY-1001 significantly improved sociability in both mice strains considered; this was once again linked to a significant improvement in NMDAR-mediated currents in the PFC. Furthermore, the authors successfully identified a group of genes related to synaptic and neuronal function, myelination, gliogenesis, and oligodendrocyte differentiation, whose expression was altered in Shank3 mice and then was improved by GSK-LSD1 treatment. Among these, further experiments identified a central role of the Egr1 gene. Ultimately, a synergetic effect between HDACi and LSD1 inhibitors was reported in adult Shank3 mice, where a significant recovery of NMDAR function in the PFC and of behavioral symptoms was observed following H3 acetylation [144].

## 8. Conclusions

The body of research here summarized suggests that many therapeutic strategies have yielded positive results for ASD core symptoms and ASD-linked cellular, anatomical, and metabolic alterations at the preclinical level. These results ultimately confirm clinical and in vitro evidence regarding the main pathways involved in ASD pathogenesis and hint at the potential for the combination of different types of treatment. The studies reviewed here showed that a treatment’s success or failure in these models usually depends on administration timing. The best results are commonly achieved when protective treatment is given in the first weeks after birth or prenatally. Unfortunately, this is not easily translatable into clinical practice as ASD diagnosis, at the moment, postdates this time window. Moreover, it is notable that most of the treatments employed in these studies did not achieve significant improvements in all the behavioral tests or definitive success in clinical trials. Despite the exact causes for the disparity between promising preclinical results and modest or negative clinical outcomes remaining unknown, a few hypotheses can be formulated. The results of many tests commonly employed to measure sociability and repetitive behaviors in mice can be altered by other symptoms known to be observed in these murine models, such as altered motor coordination, cognitive impairment, and anxiety, which may lead scientists to overestimate the effect of certain treatments on social behavior. Moreover, poor translatability may also be ascribed to the heterogeneity in symptoms and genetic backgrounds found in ASD human patients which, conversely, is far more limited in these mice strains. Ultimately, other possible confounding factors such as interactions with concurrent medications, socio-economic elements, patient lifestyle, or concomitant diseases are significantly more frequent and variable in the human population. Poor translatability may be potentially alleviated by precision medicine approaches in clinical practice and by preclinical testing of single treatments in a variety of ASD murine models. Ultimately, the present literature shows that, despite the limited clinical translational success, murine models can be a valuable tool for testing a variety of treatments in ASD research.

## Figures and Tables

**Table 1:** Table summarizing the gene or genetic loci altered in the most common genetic murine models for ASD (right) and the number of studies included in this review for each mouse model. This table does not include the number of studies employing idiopathic murine models for ASD such as VPA treated mice or BTBR mice.

Genetic Alteration	Number of Studies Reviewed
16p11.2 deletion	1
Actin like 6B (Actl6b)	/
Activity dependent neuroprotector homeobox (Adpn)	/
Activity dependent neuroprotector homeobox (Ambra1)	/
Ankyrin repeat and sterile alpha motif domain containing 1B (Anks1b)	/
Rho GTPase activating protein 32 (Arhgap32)	/
Rho guanine nucleotide exchange factor 10 (Arhgef10)	/
AT-rich interaction domain 1B (Arid1b)	/
ASH1 like histone lysine methyltransferase (Ash1l)	/
ATPase phospholipid transporting 8A1 (Atp8a1)	/
Ataxin1 (Atxn1)	/
Brain and Muscle ARNT-Like 1 (Bmal1)	2
Arginine vasopressin receptor 1B (Avpr1b)	/
Cell cycle associated protein 1 Caprin1	/
Coiled coil and C2 domain containing 1A (Cc2d1a)	/
Chromodomain helicase DNA binding protein 2 (Chd2)	/
Chromodomain helicase DNA binding protein 8 (Chd8)	/
Capicua transcriptional repressor (Cic)	/
Contactin associated protein 2 (Cntnap2)	3
Cullin 3 (cul3)	1
DEAD-box helicase 3 X-linked (Ddx3x)	/
Disco interacting protein 2 homolog A (Dip2a)	/
DLG associated protein 1 (Dlgap1)	/
Engrailed homeobox 2 (En2)	/
Fibroblast growth factor 17 (Fgf17)	/
Forkhead box P2 (Foxp2)	/
Gamma-aminobutyric acid type A receptor subunit beta3 (Gabrb3)	/
Integrin subunit beta 3 (Itgb3)	/
Lysine methyltransferase 5B (Kmt5b)	/
Methyl-CpG binding protein 2 (Mecp2)	1
Fragile X messenger ribonucleoprotein 1 (Fmr1)	7
MET proto-oncogene, receptor tyrosine kinase (Met)	/
neuroligin 3 (Nlgn3)	1
Neuronal differentiation 2 (Neurod2)	/
Neuronal growth regulator 1 (Negr1)	/
opioid receptor mu 1 (Oprm1)	1
Oxytocin receptor (Oxtr)	2
Protocadherin 19 (Pcdh19)	/
Pogo transposable element derived with ZNF domain (Pogz)	/
Phosphatase and tensin homolog (Pten)	2
RAB39B, member RAS oncogene family (Rab39b)	/
Reelin (reln)	/
Bifunctional polyamine/amino acid permease SAM3 (Sam3)	/
Sodium voltage-gated channel alpha subunit 2 (Scn2a)	/
SUMO specific peptidase 1 (Senp1)	/
SET domain containing 5 (Setd5)	/
SH3 and multiple ankyrin repeat domains 2 (Shank2)	3
SH3 and multiple ankyrin repeat domains 3 (Shank3)	14
TAO kinase 2 (Taok2)	/
T-box brain transcription factor 1 (Tbr1)	1
Ubiquitin protein ligase E3A (Ube3a)	/
Urocortin 3 (Ucn3)	/
UPF2 regulator of nonsense mediated mRNA decay (Upf2)	/
UPF3B regulator of nonsense mediated mRNA decay (Upf3b)	/
Solute carrier family 30 member 3 (ZnT3)	1

## Data Availability

Not applicable.
